# Cells that survive acute SARS-CoV-2 infection contribute to inflammation and lung regeneration in mice

**DOI:** 10.1128/mbio.03693-24

**Published:** 2025-01-29

**Authors:** Ruangang Pan, David K. Meyerholz, Stanley Perlman

**Affiliations:** 1 Department of Microbiology and Immunology, University of Iowa, Iowa City, Iowa, USA; 2 Department of Pathology, University of Iowa, Iowa City, Iowa, USA; 3 Department of Pediatrics, University of Iowa, Iowa City, Iowa, USA; Monash University, Clayton, Victoria, Australia

**Keywords:** coronavirus, SARS-CoV-2, lung regeneration, AT1 cells, AT2 cells, PATS

## Abstract

Post-acute sequelae of COVID-19 involves several organs, but its basis remains poorly understood. Some infected cells in mice survive the acute infection and persist for extended periods in the respiratory tract but not in other tissues. Here, we describe two experimental models of severe acute respiratory syndrome coronavirus-2 (SARS-CoV-2) infection to assess the effect of viral virulence on previously infected cells. Both approaches use lineage tracking of previously infected cells. In mice infected with a highly pathogenic mouse-adapted SARS-CoV-2, alveolar type 2 cells (AT2) but not alveolar type 1 (AT1) cells survived the acute infection. These cells became activated, differentiated into an AT2-to-AT1 transitional cell state (KRT8^+^ pre-alveolar type 1 transitional cell state). Additionally, nearby uninfected AT2 cells upregulated the transitional marker KRT8, thereby contributing to lung regeneration. In mice sensitized to infection by transduction with Ad5-hACE2, the infection is nonlethal, and AT1 cells survived the infection. Consequently, recovery in these mice was more rapid. Taken together, these results provide an explanation for how SARS-CoV-2 virulence contributes to poor outcomes and affects clinical recovery and lung regeneration. We also identified a new mechanism by which SARS-CoV-2 impacts lung recovery, even at times when infectious virus cannot be detected.

**IMPORTANCE:**

A major consequence of the COVID-19 pandemic is that many survivors have long-term sequelae, which are not well understood. These involve many organs, with the respiratory tract being a common site of long-term effects. Many of these sequelae can be found in mice infected with severe acute respiratory syndrome coronavirus-2 (SARS-CoV-2). In this study, we have focused on the lungs, with particular interest in the fate and role of cells that were infected with SARS-CoV-2 and survived the acute infection. We found that some infected cells survive acute SARS-CoV-2 infection and that these surviving cells both contribute to the immune response in the lungs and are involved in lung recovery. These findings illustrate previously unexplored aspects of recovery from SARS-CoV-2 induced pneumonia and may be relevant for understanding aspects of post-acute sequelae of COVID-19.

## INTRODUCTION

The ongoing COVID-19 pandemic, caused by severe acute respiratory syndrome coronavirus-2 (SARS-CoV-2), has resulted in 776,386,491 cases reported to the WHO with 7,067,260 associated deaths (as of 10 July 2024, http://covid19.who.int). Long-term sequelae have occurred in many COVID-19 survivors, especially those with severe acute disease (1–3). These long-term sequelae, called post-acute sequelae of COVID-19 (PASC) involve many organs including the cardiovascular, pulmonary, gastrointestinal, and neurological systems. PASC differs from patient to patient and is often debilitating. Several large studies have defined symptoms and signs of disease associated with PASC, but the basis of this syndrome remains unknown (1, 4, 5). Autopsy results have been inconsistent. Most studies find no evidence of viral products, but some report viral RNA (6), or less commonly, viral protein in tissues of infected patients (7). For example, some studies have reported the presence of the spike (S) protein in the blood at early times after infection (8), which could trigger long-term inflammatory responses. Alternatively, SARS-CoV-2-infected cells may be present in many tissues at early times after infection, with subsequent virus clearance but not death of the infected cell. Transient infection is difficult to assess because patient lung samples are difficult to obtain at early times after infection. This type of transient infection could induce long-term inflammatory effects, via a “hit and run” mechanism. In a similar vein, the role of viral RNA or protein in patients with PASC is difficult to investigate directly, because there are few autopsy reports of patients who died from PASC.

Because it is so difficult to study the mechanisms of PASC in humans, the development of useful experimental animal models is essential. Hamsters and nonhuman primates, among other animals, can be infected with SARS-CoV-2. Hamsters develop mild disease during the acute phase, but also show signs of long-term sequelae over several months (9). Mice cannot be directly infected with ancestral strains of SARS-CoV-2 because of incompatibility between the S protein and mouse angiotensin-converting enzyme 2 (ACE2). Several approaches have been described to sensitize mice to infection with these virus strains, including transduction with adenovirus encoding human ACE2 (hACE2) (Ad5-hACE2) (10), providing hACE2 transgenically (e.g., K18-hACE2 Tg mice) (11, 12) and mutating the virus so that it can use mouse ACE2 to enter cells (13, 14). Using the third approach, we used reverse genetics to introduce a single mutation into the virus that allowed it to use mouse ACE2 for cell entry (13). We passaged the resulting virus through the lungs to isolate a virus that was virulent in mice (SARS2-N501Y_MA30_) (15).

PASC-like signs have been identified in mice and hamsters after SARS-CoV-2 infection (9, 16), but the mechanisms contributing to these sequelae are not well understood. Specifically, it is not known whether infected cells survive the acute infection and contribute to pathogenic changes. To address this issue in mice, we used a lineage tracing approach as recently described (17, 18). We engineered recombinant SARS-CoV-2 that dually expressed Cre recombinase and Venus fluorescent protein and infected transgenic Ai9 mice, which express a floxed version of tdTomato. Cells that were acutely infected with the virus were Venus-positive, while cells that survived the infection expressed tdTomato but not Venus. As reported previously, we showed that very few, if any, previously infected cells were present in any tissue except the nasal cavity and lower respiratory tract (18). Here, we examined the fate of these surviving cells in the respiratory tract in the context of severe and mild SARS-CoV-2 infection. We found that the numbers and types of surviving cells depended on virus virulence and correlated with clinical outcomes.

## RESULTS

### Prolonged survival of previously infected cells in the respiratory tract of Ad5-hACE2-transduced mice after infection with rSARS2-WH-V2C

**Fig 1:**
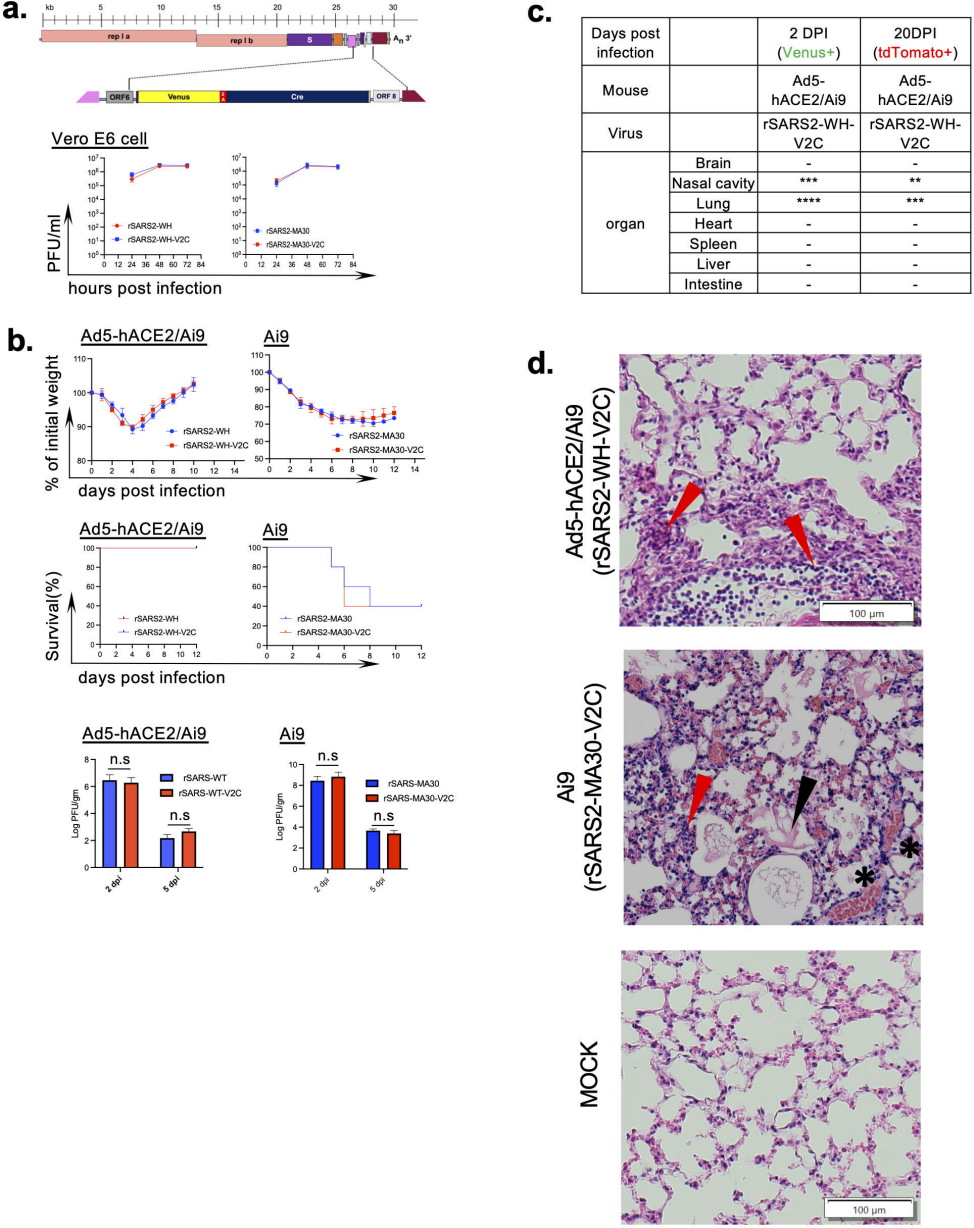
Construction and characterization of recombinant SARS-CoV-2 encoding Venus and cre proteins. (a) Schematic diagram of recombinant SARS-CoV-2 harboring Venus and CRE protein. The ORF7a gene in SARS-CoV-2 genome was replaced with Venus and Cre protein linked with a 2A peptide. Single step growth curves of rSARS2-MA30 and rSARS2-MA30-V2C (left), rSARS2-WH and rSARS2-WH-V2C (right) in Vero E6 cells. Cells were infected at an MOI of 0.01 and titered at the indicated times. (b) Three- to four-month Ai9 mice were transduced with 2.5 × 10^8^ Ad5-hACE2 (Ad5-hACE2/Ai9). Five days after transduction, transduced mice were infected with 5 × 10^4^ PFU rSARS-2-WH-V2C or rSARS-2-WH (left, *n* = 5). Three- to four-month Ai9 mice were infected with 2,000 pfu rSARS2-MA30 or recombinant rSARS2-MA30-V2C (right, *n* = 5). Mice were monitored for percentage starting weight and survival. Virus titer in the lungs was assessed on days 2 and 5 post-infection (*n* = 3). (c) Table shows sites of infection at 2 and 20 days after rSARS2-WH-V2C infection of Ad5-hACE2-transduced mice. **Five to 20 infected or surviving cells/slide, cells were observed in all mice. ***Twenty to 100 infected or surviving cells/slide, cells were observed in all mice . ****More than 100 infected or surviving cells/slide, cells were observed in all mice . (d) Pathological changes in lungs of Ad5-hACE2 transduced Ai9 mice infected with rSARS2-WH-V2C, Ai9 mice infected with rSARS2-MA30-V2C and mock-infected mice at 4 dpi. Black arrowheads, hemorrhage; red arrowheads, perivascular infiltrates; and asterisks, edema. Scale bar = 100 µm.

To determine the role in pathogenesis of cells that survive the acute SARS-CoV-2 infection, we engineered two previously described viruses (rSARS2-WH-V2C and rSARS2-MA30-V2C) (18), which express Venus fluorescent protein and Cre recombinase on backgrounds of ancestral Wuhan-Hu-1 virus or mouse-adapted virus (SARS2-N501Y_MA30_) as described above. The Venus and Cre genes were linked by a viral self-cleaving peptide, P2A, derived from porcine teschovirus-1, resulting in Cre and Venus expression after infection (Fig. 1a, upper panel). The Venus-P2A-Cre fragment (V2C) was then introduced into the ORF7 of ancestral SARS-CoV-2 or SARS2-N501Y_MA30_ genome. As expected, the replication kinetics of recombinant viruses expressing Venus and Cre was identical to that of recombinant wild-type viruses (Fig. 1a, lower panel). These results also confirmed that ORF7 was not essential for *in vitro* replication of SARS-CoV-2 (19, 20). These viruses were used to infect mice expressing a floxed tdTomato (Ai9 mice) so that any cells that survived the infection were permanently labeled by tdTomato while acutely infected cells expressed Venus. We previously identified surviving cells after infection with virulent SARS-CoV-2 (rSARS2-MA30-V2C infection of Ai9 mice and rSARS2-WH-V2C infection of Ai9- K18-hACE2 mice) (18). Here, to enable comparison with a mild SARS-CoV-2 infection, we infected Ad5-hACE2 transduced Ai9 mice with rSARS2-WH-V2C. As expected after Ad5-hACE2 transduction and infection, all mice survived the acute infection (Fig. 1b, left panel). On the other hand, 60% of 3- to 4-month Ai9 mice infected with a sublethal dose (2,000 PFU) of rSARS2-MA30-V2C succumbed to the infection (Fig. 1b, right panel). Surviving cells were detected only in the respiratory tract of either rSARS2-WH-V2C-infected Ad5-hACE2 transduced Ai9 mice (Fig. 1c) or rSARS2-MA30-V2C-infected Ai9 mice (18) when mice were analyzed at 20 dpi. Pathological analysis revealed mild edema in Ad5-hACE2 transduced Ai9 mice (Fig. 1d, left panel), but no hemorrhage, perivascular infiltrates, or severe edema, which are observed in mice with severe disease (Fig. 1d, middle panel) (12, 18, 21, 22).

### Differential survival of AT1 and AT2 cells in the lungs of rSARS2-WH-V2C-infected Ad5-hACE2-transduced Ai9 mice versus rSARS2-MA30-V2C-infected Ai9 mice

**Fig 2:**
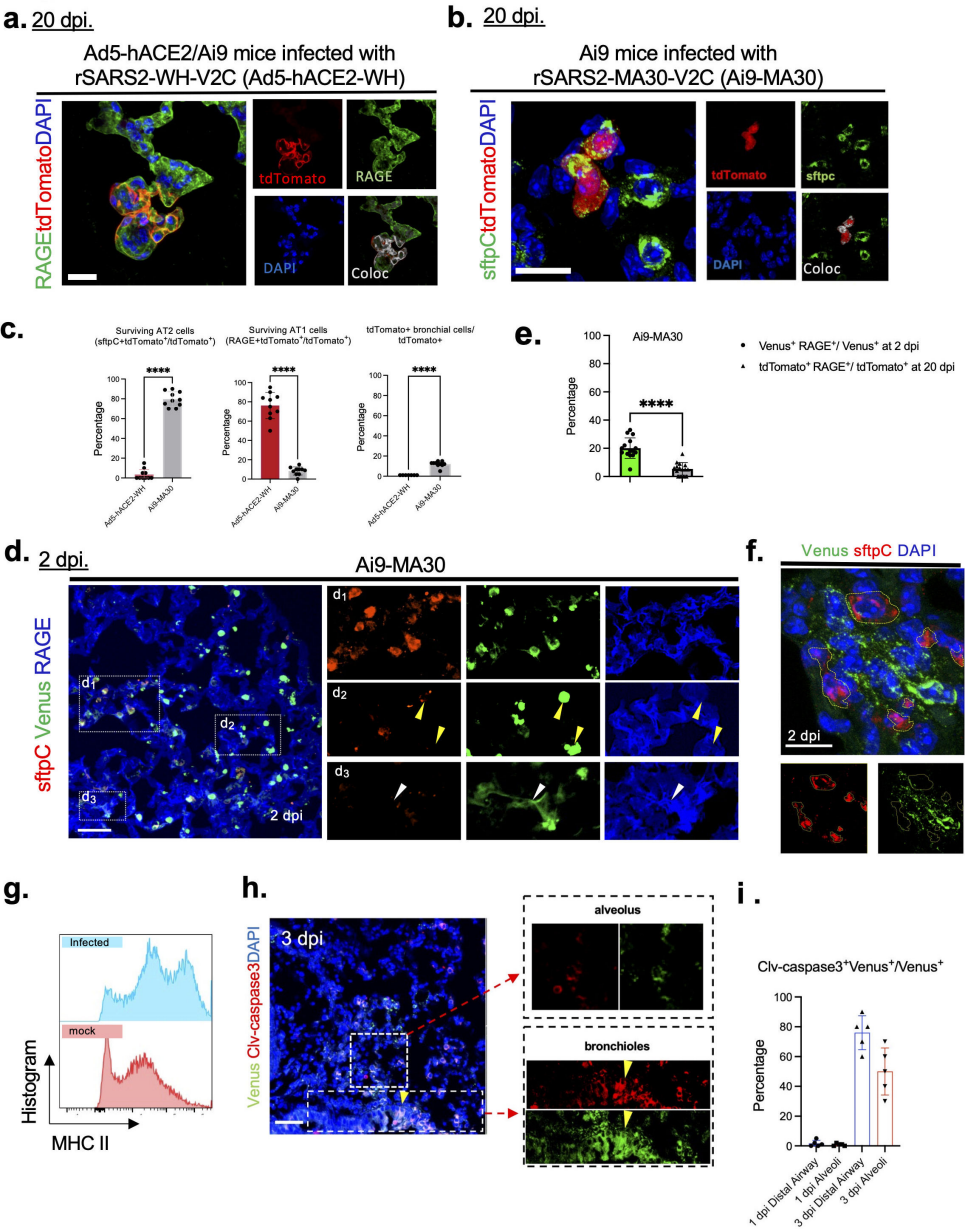
AT2 cells are immunoreactive in rSARS2-MA30-V2C-infected Ai9 mice. (a) Lung sections from rSARS2-WH-V2C-infected Ad5-hACE2 transduced Ai9 mice were analyzed for expression of tdTomato and an AT1 marker (RAGE, green) at 20 dpi. Colocalization of RAGE and tdTomato was analyzed using Fiji ImageJ as described in Materials and Methods. Scale bar, 30 µm. (b) Sections from lungs of Ai9 mice infected with rSARS2-MA30-V2C were stained for an AT2 cell marker (sftpC, green). Images of tdTomato^+^ alveolar type II (AT2) cells at 20 dpi are shown. Colocalization of sftpC and tdTomato was analyzed using Fiji ImageJ as described in Materials and Methods. Scale bar, 20 µm. (c) Percentage of surviving AT1 (RAGE^+^tdTomato^+^), surviving AT2 (sftpc^+^tdTomato^+^) and surviving bronchiolar cells (tdTomato^+^ cells localized in bronchioles) of total tdTomato^+^ cells in the lungs of Ad5-hACE2-transduced Ai9 mice infected with rSARS2-WH-V2C (red), or Ai9 mice infected with rSARS2-MA30-V2C (gray) at 20 dpi. *n* = 5 mice/group, *n* > 3 sections/mouse. Statistical analysis was performed using two-tailed unpaired, non-parametric *t* test (Mann-Whitney test); ****P < 0.0001. (d) Images of Venus^+^ AT1 (RAGE^+^) or AT2 cells (sftpC^+^) in Ai9 mice infected with rSARS2-MA30-V2C at 2 dpi. Higher magnification images of selected areas are shown (d_1_, d_2_, and d_3_). Yellow arrowheads in panel d_2,_ 40× (right panel) indicate AT2-like cuboidal Venus^+^sftpC^low/negative^ cells. White arrowheads in panel d_3,_ 40× (right panel) indicate a Venus^+^RAGE^+^ cell. Scale bar, 50 µm. (e) Percentage of infected AT1 cells within total Venus^+^ cell population (RAGE^+^Venus^+^/Venus^+^) at 2 dpi and surviving AT1 cells within total tdTomato^+^ cell population (RAGE^+^tdTomato^+^/tdTomato^+^) at 20 dpi in lungs of Ai9 mice infected with rSARS2-MA30-V2C. (f) AT2 cells (stfpC^+^) in areas with extensive virus infection were minimally infected (Venus^+^). Clusters of AT2 cells (sftpC) are outlined. Scale bar, 10 μm. (g) MHC II expression on total epithelial cells (CD45^−^Epcam^+^, gating strategy is shown in Fig. S1b) was analyzed by flow cytometry. Upregulated MHC II expression on epithelial cells was observed at 2 dpi compared to mock. (**h and i**) Sections were stained for cleaved-caspase 3 expression in lungs at 3 dpi. Fraction of Venus^+^ cells that were positive for cleaved-caspase 3 is summarized in panel h. Yellow arrowheads indicate an airway (bronchiole). Scale bar, 50 µm. Higher magnification images are shown in panels d, e, and g.

Our previous results showed that the main site of surviving, previously infected cells was the lung (18). The majority of surviving cells were located in the lung parenchyma, with only occasional surviving cells in the airway (18). We observed striking differences in the types of surviving lung cells when mice with very mild infection (Ad5-hACE2-transduced) were compared to those infected with rSARS2-MA30-V2C (Fig. 2a through c). Surviving cells in the lungs of Ad5-hACE2-transduced mice at 20 days post-infection (dpi) were predominantly mature type 1 alveolar (AT1) cells (RAGE^+^), had normal AT1 morphology and were expected to be functional for gas exchange (Fig. 2a and c). In contrast, over 80% of the surviving cells in the lung parenchyma of rSARS2-MA30-V2C-infected Ai9 mice were type 2 alveolar (AT2) cells (SFTPC^+^) (Fig. 2b), with occasional AT1 cells observed (Fig. 2c). When comparing lung tissues at 20 dpi to those at 2 dpi, we found a significant reduction in the number of surviving AT1 cells (Fig. 2e). In some mice, almost no surviving AT1 cells were detected in the lungs. Approximately 5–15% of the surviving cells were detected in the bronchioles of Ai9 mice after rSARS2-MA30-V2C infection (Fig. 2c). Since AT1 cells appeared to survive in rSARS2-WH-V2C-infected Ad5-hACE2-transduced Ai9 mice, this demonstrates that AT1 cell infection is not necessarily lethal in mice. However, it should be noted that hACE2 expression via Ad5 transduction is artificial, limiting the utility of this approach for studies of pathogenesis. Furthermore, this observation was inconsistent with reports from COVID-19 patients, where AT2 cells are identified as the primary target cells of SARS-CoV-2 infection in humans (23, 24). Therefore, we analyzed rSARS2-MA30-V2C-infected Ai9 mice for the remainder of this study.

Venus expression was prominent in the lungs of all infected mice at 2 dpi but was significantly decreased by 5 dpi. While infected AT1 (RAGE^+^) and AT2 (pro-surfactant protein C (sftpC)^+^) cells were detected at early times in rSARS2-MA30-V2C-infected Ai9 mice (Fig. 2d), most were AT2 cells (sftpC^+^Venus^+^) (Fig. 2d1), consistent with previous reports (23, 24). Occasional infected AT1 cells also were detected (RAGE^+^Venus^+^) (Fig. 2d3, white arrowheads). Interestingly, we observed a subset of cells with high Venus expression that exhibited classic cuboidal AT2 cell morphology but expressed only very low or nil levels of sftpC (Fig. 2d2, yellow arrowheads). This result is in agreement with previous reports showing that acute SARS-CoV-2 infection resulted in the loss of sftpC expression by AT2 cells in both human and murine infections (21, 24, 25). Notably, in some areas of extensive viral infection, nearby AT2 cells appeared to be minimally infected (Fig. 2f). This may occur because AT2 cells are known to be immunologically active (e.g., these cells express types I and III interferon after SARS-CoV-2 infection [26]), which potentially provides some protection against acute infection. In support of this notion, we found that infected compared to control cells expressed higher levels of MHC II at 2 dpi. As MHC II is expressed by AT2 but not AT1 cells, higher MHCII^hi^ expression levels are suggestive of enhanced antigen presentation and immune-responsiveness. (Fig. 2g; Fig. S1).

Since the number of surviving cells appeared to be much lower than the number of infected cells, we next analyzed lungs for evidence of apoptosis during the acute phase. We detected widespread areas of apoptosis throughout the airway and lung parenchyma at 3 dpi (Fig. S2a; Fig. 2i). A higher percentage of Venus^+^ cells in the distal airway compared to the alveoli had evidence of apoptosis (Fig. 2h1), resulting in decreased numbers of surviving cells in the airway (Fig. S2b).

### AT2 cells differentiate into AT1 cells in rSARS2-MA30-V2C-infected Ai9 mice

**Fig 3:**
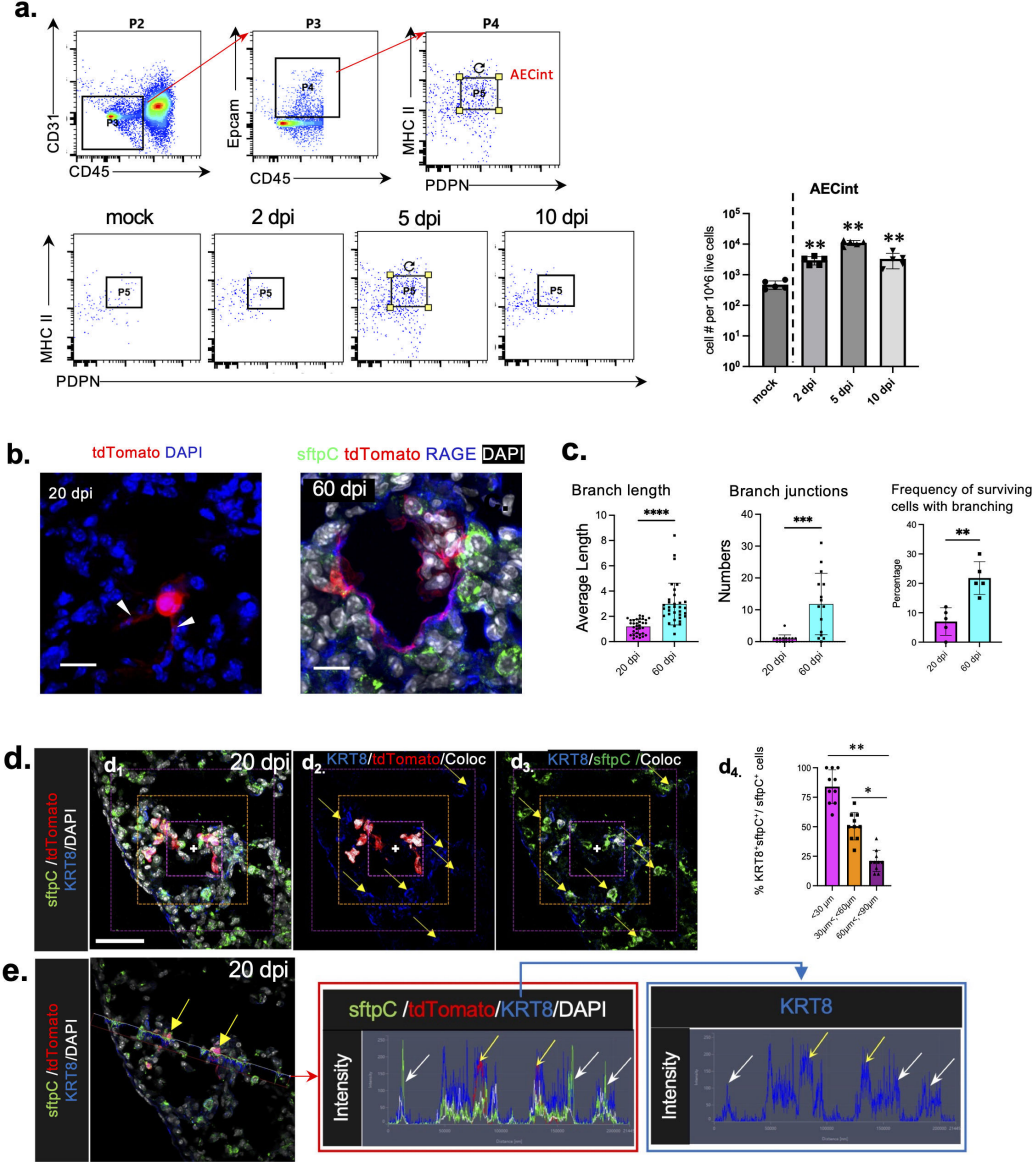
Previously infected and neighboring bystander AT2 cells are activated. (a) AECint cells (CD45^−^CD31^−^Epcam^+^PDPN^hi^) were analyzed by flow cytometry following infection. The gating strategy for AECint cells is shown in the upper panel. Representative images of AECint cells at different times p.i. are displayed in the lower left panel, with a summary of the data shown in the lower right panel. Statistical analyses (2 dpi vs mock, 5 dpi vs mock, and 10 dpi vs mock) were performed using two-tailed unpaired, non-parametric *t* tests (Mann-Whitney test); *P < 0.05 and **P < 0.01. (**b**) Sections from lungs of rSARS2-MA30_-_V2C-infected Ai9 mice were analyzed for tdTomato expression at 20 dpi (left panel) and 60 dpi (right panel). Single channels for tdTomato (red), sftpC (green), and RAGE (blue) are shown in Fig. S3. Scale bar, 20 µm. (c) Summary of the skeleton length (left), number of junctions of tdTomato^+^ cells (middle), and percentage of tdTomato^+^ cells with Junctions within total tdTomato^+^ cells population at 20 and 60 dpi (*n* = 5 samples per group, *n* = 5–10 slides per sample). Statistical analysis was performed using two-tailed unpaired, non-parametric *t* test (Mann-Whitney test); ***P < 0.001 and ****P < 0.0001. (d) Sections from lungs of rSARS2-MA30-V2C-infected Ai9 mice were analyzed at 20 dpi for DAPI (white), sftpC (green), KRT8 (blue), and tdTomato (red). Boxed area in panel d_1_ was further analyzed in panels d_2_ (colocalization of tdTomato/KRT8) and d_3_ (colocalization of sftpC/KRT8). KRT8^+^sftpC^+^ bystander cells were located in the vicinity of previously infected tdTomato^+^ cells; numbers decreased with distance from tdTomato^+^ cells. Colocalization was analyzed using ImageJ and percentage of KRT8^+^ AT2 cells/AT2 cells in regions of interest (ROIs) is summarized in panel d_4_. Yellow arrowheads in panels d_2_ and(d_3_ indicate sftpc^+^KRT8^+^ tdTomato cells. The white cross in the center of panels d_1_ to d_3_ represents the central area of the image and ROIs. Scale bar, 50 µm. (e) Lung sections from rSARS2-MA30-V2C-infectd Ai9 mice were stained as in panel **d**. (Right) Cells along the indicated line (left panel) were analyzed for marker expression using ZEN software (Zeiss). In brief, the *x*-axis in the middle and right-hand panels represents the localization of the protein along the axis shown in the left-hand panel, while the *y*-axis represents the expression intensity of the indicated protein at each location. The yellow arrows indicated cells with high expression of tdTomato, and the white arrows indicated cells with high expression of sftpC. Higher expression of KRT8 in tdTomato^+^ (yellow arrows) compared to tdTomato^−^sftpc^+^ (white arrows) cells is shown.

To assess whether surviving previously infected AT2 are functional, we next examined their ability to differentiate into AT1 cells. AT2 cells serve as precursors to AT1 cells (27) by transitioning through a pre-alveolar transitional cell state (PATS). In specific, CD45^−^Epcam^+^PDPN^high^MHCII^high^ termed “intermediate alveolar epithelial cells” (AECint) (28), increased during recovery after lung injury induced by influenza A virus infection. We analyzed the lungs of rSARS2-MA30-V2C-infected Ai9 mice for the presence of AECint. We found that AECint cells expanded very efficiently after infection, reaching their peak at 5 days post-infection and decreasing by 10 dpi (Fig. 3a). This indicated that AT2 cells were activated early during SARS-CoV-2 infection, initiating the transition to AT1 cells. We also examined infected lungs for evidence of a precursor-product relationship by examining tdTomato^+^ cells for phenotypic changes consistent with AT2 to AT1 cell conversion at 20 and 60 dpi. Unlike AT2 cells which are cuboidal, AT1 cells are elongated and have extensive branching. We detected a small number of branches on previously infected tdTomato^+^ cells at 20 dpi (Fig. 3b, left panel, arrowheads), suggesting that they were in the initial process of transitioning (29, 30). By 60 dpi, some tdTomato^+^ cells were RAGE^+^ SFTPC and displayed an AT1 cell-like morphology (more elongated, thin, and flat) (Fig. 3b, right panel; Fig. S3a), suggesting that these activated AT2 cells had completed the transition. Because branching increases during AT2 to AT1 cell transition, we next quantified the number and length of branches (Fig. S4a) on tdTomato^+^ cells, as well as the complexity of the branches by counting the numbers of junctions. There were significant increases in cell (skeleton) length and numbers of skeleton junctions at 60 dpi compared to 20 dpi (Fig. 3c)**,** also suggestive of AT2 to AT1 transition.

Previous studies identified a pre-AT1 cell (PATS) that originated from AT2 cells after acute bleomycin lung injury or diphtheria toxin-mediated AT1 depletion and expressed keratin-8 (KRT-8) (31, 32). We found that KRT-8 was highly expressed by most surviving AT2 cells at 20 dpi in rSARS2-MA30-V2C-infected lungs (Fig. 3d1 and d3). Additionally, KRT-8 expression was preferentially upregulated in uninfected cells in the vicinity of clusters of infected cells (Fig. 3d4), with expression diminishing with distance from surviving cells (Fig. 3e), suggesting that surviving AT2 cells contributed to alveolar regeneration mediated by uninfected AT2 cells.

### Differentiation of surviving AT2 cells is inhibited during prolonged phase

**Fig 4:**
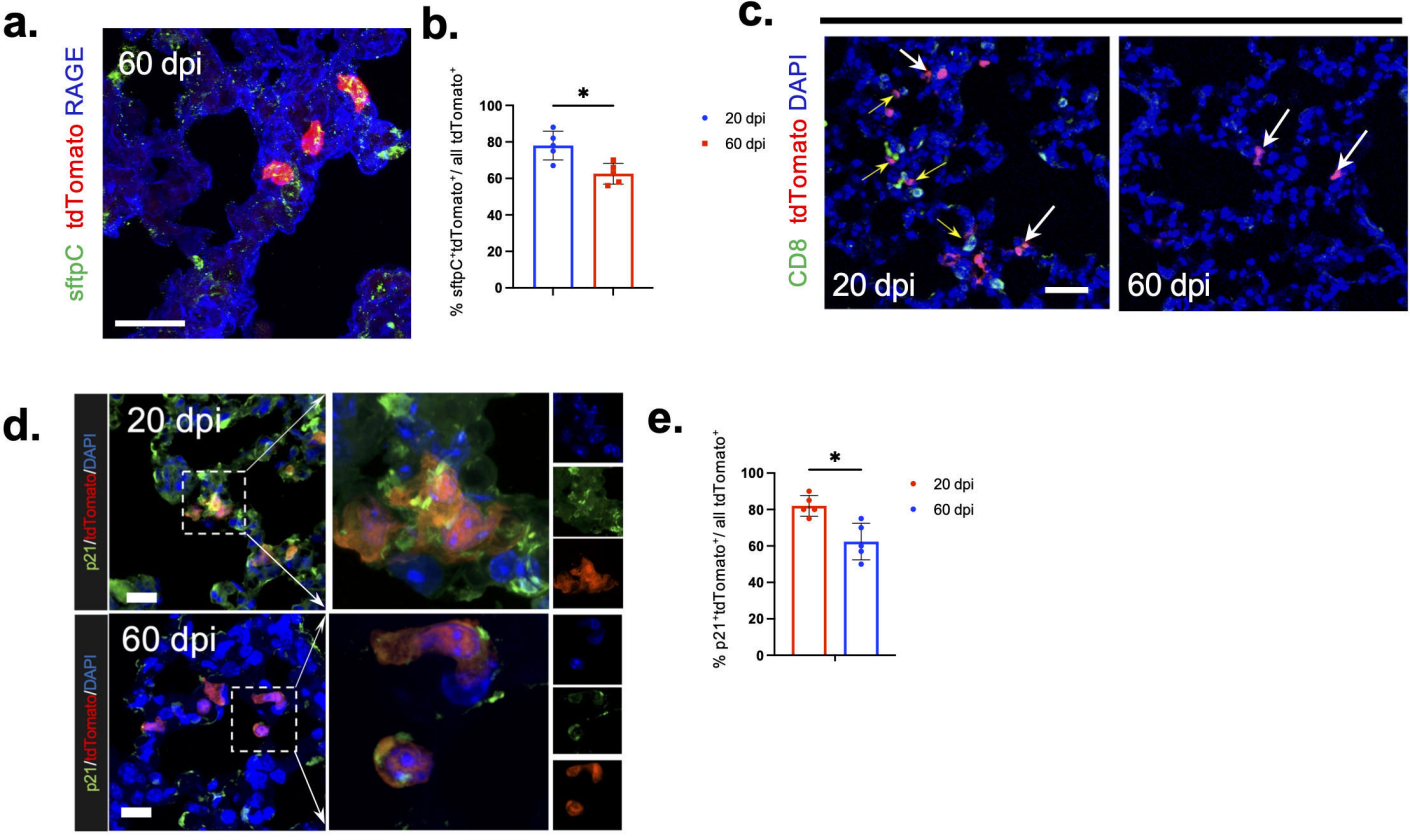
Expression of CDKN1A (p21) in previously infected cells at 20 and 60 dpi. (a) Sections from lungs of from rSARS2-MA30-V2C-infected Ai9 mice were stained for sftpC expression at 60 dpi. Scale bar, 20 µm. (b) Fraction of sftpC^+^tdTomato^+^ cells of all tdTomato^+^ is shown at 20 and 60 dpi. *n* = 5 mice. (c) Sections from lungs of from rSARS2-MA30-V2C-infected Ai9 mice were stained for CD8 expression at 20 dpi and 60 dpi. Surviving cells interacting with CD8 T cells (yellow arrows) or not interacting with CD8 T cells (white arrows) are indicated. Scale bar, 50 µm. (d) Sections from lungs of rSARS2-MA30-V2C-infected Ai9 mice were stained for p21 (green) at 20 and 60 dpi. Higher magnification images of selected areas in left are shown. Scale bar, 20 µm. (e) Portion of p21^+^ and tdTomato^+^ cells in all tdTomato^+^ cells were summarized. *n* = 5 mice. Statistical analysis was performed using two-tailed unpaired, non-parametric *t* test (Mann-Whitney test); *P < 0.05.

Although most of these surviving cells expressed KRT8, suggesting they were en route to an AT2-AT1 transitional state (31), over 50% of these cells did not successfully mature into AT1 cells by 60 dpi (Fig. 4a and b). These cells failed to differentiate fully, suggesting they remained in a transitional state. CD8^+^ T cells were recently shown to contribute to maintaining this transitional state through interactions with macrophages, leading to impaired alveolar regeneration (33). We also observed more CD8 T cells in the vicinity of tdTomato^+^ cells at 20 dpi, consistent with interactions between the two cell types (Fig. 4c, left panel, yellow arrow indicated), while other CD8 T cells were not in close vicinity (Fig. 4c, left panel, white arrows). These cells were also CD3^+^ further indicating that they are CD8 T cells (Fig. S3). However, by 60 dpi, we detected fewer CD8 T cells in the vicinity of prior infected cells and little evidence of interaction between tdTomato^+^ and CD8 T cells (Fig. 4c, right panel, white arrows). Despite this, the surviving cells still exhibited an AT2 morphology, suggesting incomplete differentiation.

Elevated levels of additional PATS markers, including Sox4, γH2AX, Cldn4, LGALS3, and CDKN1A/p21, have been described in settings of lung injury caused by bleomycin exposure or human and mouse SARS-CoV-2 infection using single-cell mRNA sequencing analysis (21, 23, 32). We extended these analyses to cells in mice that survived acute SARS-CoV-2 infection, using immunofluorescence analysis to detect PATS-associated proteins. CLDN4 and LGALS3 were previously reported to be expressed during AT2 to AT1 cell transition in lung injury models using organoids and mice, with rare expression in mature AT1 cells (32). We found that Cldn4 and LGALS3 were slightly upregulated in surviving cells and in the vicinity of these cells at 20 dpi (Fig. S5a, summarized in Fig. S5b); however, the expression of these two markers was diminished at 60 dpi, even though AT2-AT1 transition was not complete (Fig. S5a, summarized in Fig. S5b). DNA damage induces the emergence of PATS cells in bleomycin-induced lung damage (32). Since SARS-CoV-2 has been shown to induce DNA damage in cell culture and patients (34–36), we next analyzed the expression of γH2AX and Sox4 (markers of DNA damage and cytoskeleton elongation following DNA damage, respectively), but neither protein was expressed in tdTomato^+^ cells (Fig. S5a, summarized in Fig. S5b). In contrast, the protein encoded by *CDKN1A*, p21, is a cell senescence marker and is expressed through p53-dependent transcriptional activation. P21 was highly expressed in surviving cells and in the vicinity of these cells at 20 dpi (Fig. 4d, upper left panel). Expression of p21 was also detected in the surviving cells at 60 dpi, although at lower levels (Fig. 4d, lower left panel, summarized in right panel).

### Relationship between surviving cells and subsequent development of fibrosis

**Fig 5:**
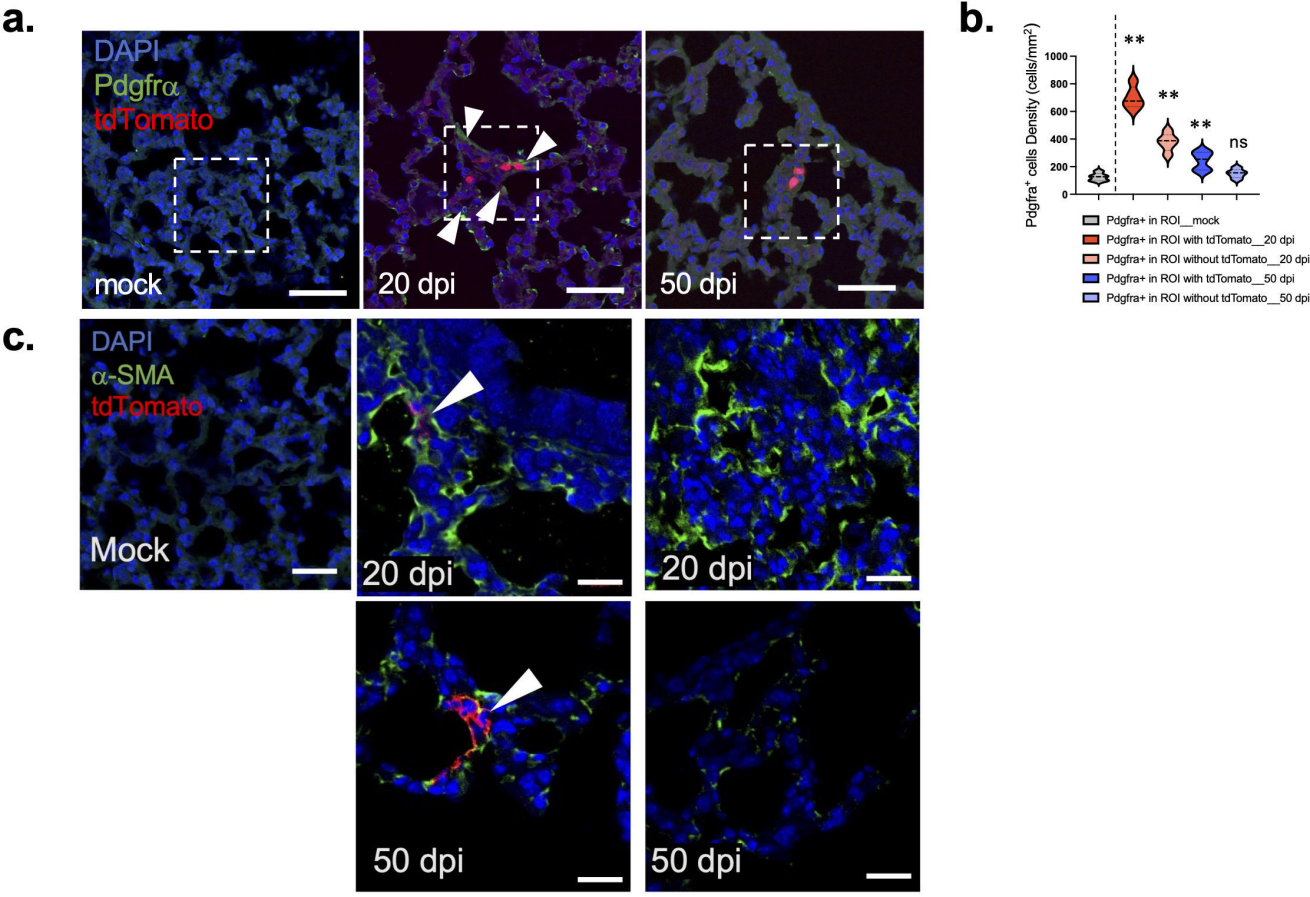
Pdgfrα^+^ fibroblasts are recruited to the vicinity of tdTomato^+^ cells at 20 dpi. (a) Sections from lungs of Ai9 mice infected with rSARS2-MA30-V2C were analyzed for expression of tdTomato and pdgfrα at 20 and 50 dpi. Scale bar, 50 µm. (b) Quantification of pdgfrα cells in region of interest (ROI; dotted lines in panel **a**) at different time points. *n* = 5 mice/group; *n* > 3 sections/mouse. Pdgfrα cells accumulated in the vicinity of tdTomato^+^ cells at 20 dpi but to a lesser extent at 50 dpi. (c) Lung sections from rSARS2-MA30-V2C-infected Ai9 mice were analyzed for expression of tdTomato (red^+^) and α-SMA (green^+^) at 20 and 50 dpi. α-SMA expression was upregulated in the vicinity of both tdTomato^+^ (middle panels) and tdTomato^−^ cells (right panels) at 20 dpi (upper panels) and decreased by 50 dpi (bottom panels) after infection. Scale bar, 50 µm. Statistical analysis was performed using two-tailed unpaired, non-parametric *t* test (Mann-Whitney test); **P < 0.01; ns, not significant.

Pdgfrα^+^ cells are reported to support the differentiation of AT2 cells within regenerative niches in adult lungs (27, 37). Ablation of Pdgfrα^+^ cells led to emphysema-like enlargement of distal airspaces during embryogenesis, resulting in defective alveolar structure and function (38, 39). Examination of SARS-CoV-2-infected lungs revealed the presence of Pdgfrα^+^ cells in the vicinity of previously infected cells at 20 dpi, with lower levels detected by 50 dpi, consistent with the possibility that these cells participate in regeneration (Fig. 5a and b).

Fibrotic changes were reported in the lungs of mice that survived acute SARS-CoV-2 infection (21). To assess whether fibrosis preferentially occurred in the vicinity of previously infected cells, we examined lungs at days 20 and 50 after staining for α-smooth muscle actin (α-SMA), a marker for fibrosis. We detected diffuse α-SMA labeling, without specific localization to sites of previously infected cells at day 20 p.i. (Fig. 5c), possibly reflecting broad damage to the lungs including at sites where all infected cells were cleared. α-SMA expression was diminished by day 50 p.i, indicating that virus-induced fibrosis had largely resolved by this time point.

### Prolonged infected cell survival in the nasal cavity of rSARS2-MA30-V2C-infected Ai9 mice

**Fig 6:**
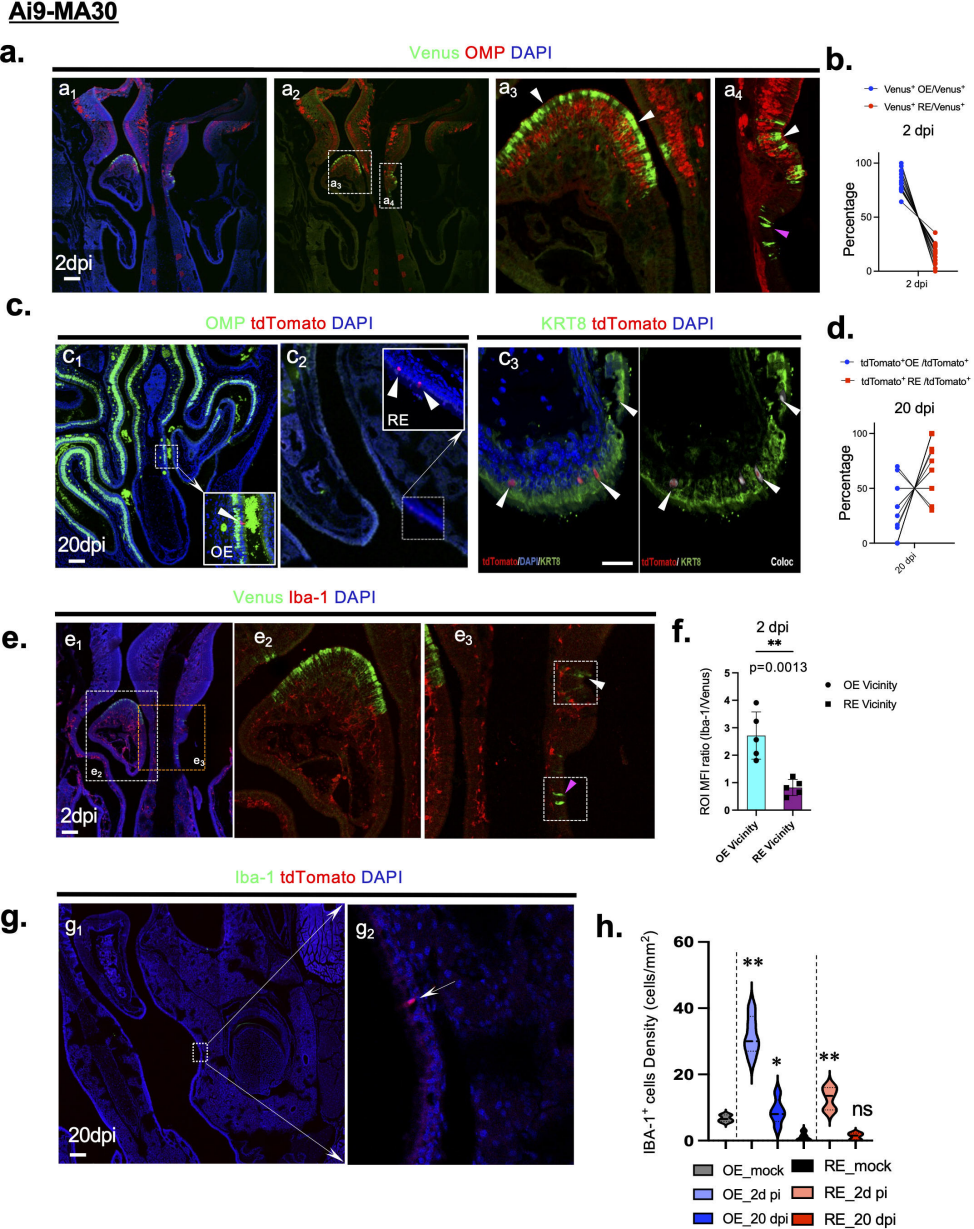
rSARS-CoV-2 infection of olfactory epithelium cells. (a) Nasal cavity sections from Ai9 mice infected with rSARS2-MA30-V2C were stained for olfactory marker protein (OMP, Red) at day 2 p.i. Venus^+^ cells (green) were detected predominantly in OMP^−^ cells in the olfactory epithelium (OE) (OMP^+^ area). Higher magnification images of selected areas in panel a_2_ are shown in panels a_3_ and a_4_. White and pink arrowheads indicate Venus^+^ olfactory epithelium (OMP^+^ area) and respiratory epithelium (RE) (OMP^−^ area), respectively. Scale bar, 100 µm. (b) Percentage of Venus^+^ cells detected in the OE compared to the RE at 2 days post-infection (dpi). A minimum of three slides per mouse and five mice per group were analyzed. (c) Nasal cavity sections from Ai9 mice infected with rSARS2-MA30-V2C were stained for OMP (green) at day 20 p.i. tdTomato^+^ cells (red) were detected in both the olfactory epithelium (OMP^+^ area, c_1_) and RE (OMP^−^ area, c_2_). Higher magnification images of selected areas in panels c_1_ and c_2_ are shown. Image of surviving KRT8^+^ (green) tdTomato^+^ (red) sustentacular cells at 20 dpi (green, c_3_. Coloclization of KRT8 and tdTomato was quantified by ImageJ (c_3_, right panel). Scale bar, 100 µm in panel **c_1_**, 50 µm in panel **c_3_**. (d) Percentage of total tdTomato^+^ cells in OE compared to the RE at 20 dpi. A minimum of three slides per mouse and five mice per group were analyzed. (**e–h**) Nasal cavity sections from Ai9 mice infected with rSARS2-MA30-V2C were stained for Iba-1. Iba-1 (red) and Venus (green) are shown at 2 dpi (e), Iba-1 (green), and tdTomato (red) are shown at 20 dpi (g). Iba-1 decreased nearly to basal levels by 20 dpi (h). Higher magnification images of selected areas in panel e_1_ are shown in panels e_2_ and e_3_. Higher magnification image of selected area in panel g_1_ is shown in panel g_2_. Fluorescent Intensity of Iba-1 expression in region of interest (ROI) surrounding Venus^+^ OE (white arrowhead) versus Venus^+^ RE (pink arrowhead) is summarized in panel f, *n* = 5 mice group, 5–10 sections/mouse; each spot in panel f represents mean value from all sections from a single mouse. Representative ROIs are identified in panel e_3_; each ROI is a 100 μm × 100 µm area surrounding infected cells in OE or RE. Quantification of Iba-1^+^ cells at 2 and 20 dpi in panel h. *n* = 5 mice per group, *n* > 3 sections per mouse. OE and RE areas were determined by the OMP expression level as shown in panel a_4_. Scale bar, 100 µm. Statistical analysis was performed comparing OE 2 dpi versus OE mock, OE 20 dpi versus OE mock, RE 2 dpi versus RE mock, RE 20 dpi versus RE mock. Statistical analysis was performed using two-tailed unpaired, non-parametric *t* test (Mann-Whitney test); *P < 0.05, **P < 0.01, ns, not significant.

Since the nasal cavity was robustly infected during the acute phase, and harbored surviving cells (18), we performed a more detailed study of this site after rSARS2-MA30-V2C-infection. In agreement with previous results (40), we found that at 2 days post-infection, the virus primarily infected cells in the olfactory epithelium (OE) (Fig. 6a1 through 6a4, white arrowhead, summarized in Fig. 6b), and in the respiratory epithelium (RE) (Fig. 6a4, pink arrowhead) located adjacent to the OE. Sustentacular cells but not olfactory sensory neurons were infected in the OE in agreement with previous results (40–42). However, by comparing the numbers of cells that were infected at 2 days p.i. (Venus^+^) to numbers that survived the infection at 20 days p.i. (tdTomato^+^), we found that only a few of the infected cells in the OE appeared to survive (Fig. 6a1 vs c1). These surviving cells were cytokeratin KRT8^+^ (Fig. 6c3), which is a sustentacular cell marker in the nasal cavity (43). We also identified surviving cells in the RE (Fig. 6c2). There was a substantial increase in the proportion of surviving cells in the RE versus OE at later times, suggesting preferential clearance of infected cells or more rapid cell turnover in the OE, (Fig. 6b vs d). In terms of the cellular immune response, we observed increased numbers of Iba-1^+^ macrophages in the rSARS2-MA30-V2C-infected nasal cavity at 2 dpi, with greater numbers of Iba-1^+^ cells in the OE compared to the RE in the vicinity of Venus^+^ cells (Fig. 6e2 through 6e3, Summarized in Fig. 6f). Iba1^+^ cell numbers nearly returned to basal levels by 20 dpi (Fig. 6e1 and g1, summarized in Fig. 6h). Taken together, these results suggest most of the infected cells in the OE do not survive the acute infection, possibly due to the enhanced antiviral response at this site.

## DISCUSSION

Here, we confirm that respiratory tract cells that were infected with rSARS-CoV-2 survive for extended periods of time after recovery from acute disease. We also show that the type of surviving cell differs depending on the method used to sensitize mice for infection and the subsequent severity of clinical disease. Mice in which hACE2 was provided by Ad5-hACE2 transduction develop very mild disease, and in these mice, AT1 cells were mainly infected and survived the acute infection (Fig. 2a). Extensive survival of infected AT1 cells likely enhanced mouse survival. This prolonged survival of infected mice and infected cells may have occurred because Ad5 transduction may induce an innate immune response able to attenuate the virus infection. Additionally, the innate immune response induced by Ad5-hACE2 transduction may have also resulted in fewer AT2 cells being infected since these cells are immunoresponsive (44). While at the level of the organism, fewer cells were infected because receptor is only expressed on transduced cells.

On the other hand, rSARS2-MA30-V2C-infected Ai9 mice developed severe pulmonary disease. In these mice, mainly AT2 cells were infected during the acute phase of the infection, although infected AT1 cells could be detected at 2 dpi (Fig. 2d), consistent with previous results (21). In contrast, single-cell RNA sequencing analyses of COVID-19 patient samples showed that markers of both AT1 (RAGE) and AT2 (SFTPC) cells were decreased during the acute phase, with continued loss of AT1 cells in patients that succumbed to the infection (24). By analogy, it is possible that AT1 cells were infected with rSARS2-MA30-V2C but did not survive the infection, resulting in the detection of only previously infected AT2 cells in lungs during convalescence. Infected AT2 may have survived the acute infection because they were immunoresponsive, expressing MHC class II. Of note, even after expressing a potentially protective immune response, only a small fraction of infected AT2 cells survived the acute phase (18). MHC class II is also upregulated on AT2 cells in the lungs of mice infected with other respiratory viruses, such as influenza A virus (IAV) and Sendai virus (SeV) (44). Deletion of MHC II class specifically from AT2 cells resulted in enhanced IAV replication and more severe clinical disease confirming the importance of AT2 cell activation in protection (44).

These previously infected AT2 cells also contributed to lung recovery, since at least some of them were able to differentiate into AT1 cells. Virtually 100% of previously infected AT2 cells expressed KRT-8 at 20 dpi, indicating that they were activated and had undergone AT2-AT1 cell transition to a variable extent. AT2 cell activation also appeared to extend to cells that had not been previously infected but were in the vicinity of the tdTomato^+^ cells as assessed by KRT-8 expression (Fig. 3d and e). This suggested that one or more soluble pro-inflammatory factors contributed to the activation of nearby AT2 cells. Such putative factors include type I and III interferons, TNF, and IL-1β (26, 45, 46).

Some AT2 cells completed differentiation to AT1 cells, as shown by phenotypic (RAGE^+^SFTPC^−^) and morphologic (elongated, thin, and flat cell shape as well as a greater number of branches of increased length) changes by 60 dpi compared to cells examined at day 20 p.i. (Fig. 3b and c**;** Fig. S3a). Other cells showed evidence of only partial differentiation, remaining SFPTC^+^. One possible explanation for these differences in differentiation is the inhibitory effect that IFN (especially IFN-III) signaling has on lung repair. IFN-III signaling induces p53, thereby hampering epithelial differentiation (47). We also detected CD8 T cells located adjacent to previously infected cells. A recent report indicated that IFNγ released from CD8 T cells stimulates interstitial macrophages and inhibits epithelial progenitor differentiation in SARS-CoV-2 or influenza A virus-infected lungs (33). However, while CD8 T cells are generally cytotoxic, human resident CD8 T cells with regenerative properties were recently described and if present in mice, could contribute to remodeling of SARS-CoV-2 infected lungs (48). The exact role of CD8 T cells during the recovery phase is not clear, but we observed that at 20 dpi, there was a greater accumulation of CD8 T cells in the vicinity of prior infected cells compared to 60 dpi, suggesting some resolution of the immune response. As shown above, most previously infected AT2 cells expressed KRT8 at 20 dpi (Fig. 3d), indicating they had initiated transition (31). Although CD8 T cells were no longer detected to a great extent at 60 dpi, many previously infected cells still had not fully differentiated into AT1 cells. This suggests that other regulatory mechanisms, independent of CD8 T cells, may persist and inhibit the differentiation of AT2 to AT1 cells. These differences in the ability to complete differentiation may impact the ability of the lung to fully regenerate in mice. Similar observations were made when SARS-CoV-2-infected human lungs were examined at autopsy (24) and may explain the slow process of lung regeneration observed in some patients (23).

We also observed upregulated expression of several proteins associated with PATS including most prominently, p21, within surviving cells and non-infected cells located in the vicinity of prior infected cells. p21 is a known downstream target of p53 and has been implicated in actively inhibiting apoptosis and in the response to DNA damage. Several reports have demonstrated SARS-CoV-2-mediated DNA damage as early as 2 dpi. However, we did not observe activation of the DNA damage marker,γH2AX, in surviving cells, possibly because DNA damage had already resolved in these surviving cells. Early DNA damage caused by viral infection may induce cellular apoptosis and upregulation of p21 expression through the p53 pathway.

Similarly, we found upregulation of PDGFRa, a myofibroblast progenitor marker, on cells in the vicinity of surviving cells at day 20 p.i., with decreased levels detected at day 50 p.i. Pdgfra^+^ cells are involved in the proliferation and differentiation of alveolar cells (29, 49), but their specific role remains unclear. In addition, Pdgfra^+^ cells serve as progenitors to several cell types, dependent on context (27, 50–52). Although myofibroblasts have been reported to enhance lung regeneration (46), recent findings suggest that these cells may also inhibit lung regeneration (53). Given that previously infected AT2 cells exhibit varying levels of differentiation, these Pdgfra^+^ cells could have diverse roles that may be context-dependent.

The other major site of surviving cells was the nasal cavity. Sustentacular cells in the OE were the predominant cell type infected in the nasal cavity (40) but previously infected cells in the RE appeared to survive to a greater extent than did sustentacular cells in the OE (Fig. 6b vs d. As was the case in lungs, the numbers of surviving cells at later times after infection were much lower than the numbers of acutely infected cells (18), suggesting that most sustentacular cells died and were cleared during the acute phase, possibly contributing to anosmia (54).

Collectively, these results suggest that in mice, these previously infected cells contribute to ongoing inflammatory responses. It will be important to determine the extent to which these cells contribute to or inhibit lung regeneration and possibly enhance the development of pulmonary PASC.

## MATERIALS AND MATERIALS

### Mice

Specific-pathogen-free C57Bl/6 mice were purchased from Charles River Laboratories. B6.Cg-*Gt(ROSA)26Sor^tm14(CAG-tdTomato)Hze^*/J (Ai9) mice were purchased from Jackson Laboratories. Twelfth- to 16-week-old male and female mice were intranasally inoculated with the indicated viruses after ketamine/xylazine anesthesia. After virus infection, mice were monitored and weighed daily. All experiments with SARS-CoV-2 were performed in a Biosafety Level 3 (BSL3) laboratory at the University of Iowa. All animal studies were approved by the University of Iowa Animal Care and Use Committee (IACUC) and meet stipulations of the Guide for the Care and Use of Laboratory Animals.

### Generation of rSARS-CoV-2 or rSARS-CoV-2-MA30 encoding Venus and cre proteins

rSARS2-MA30-V2C and rSARS2-WH-V2C were constructed as previously described (18).

### Rescue of SARS-CoV-2 or SARS-CoV-2-MA30 encoding the Venus and cre proteins

Confluent monolayers of Vero E6 cells overexpressing hACE2 and TMPRSS2 (A2T2 Cells) (10^6^ cells per well, six-well plates) were transfected with 2.0 ug per wells of SARS-CoV-2 BAC using Lipofectamine 3000. Cells were monitored daily for cytopathic effects (CPEs). Cultures were collected when the CPE was >50% and frozen at −80°C. Recombinant viruses were further passaged in A2T2 cells in DMEM supplemented with 10% fetal bovine serum (FBS). Cultures were collected when the CPE was >50% and frozen at −80°C. Virus titers were determined by plaque assay.

### Plaque assay

Twelve-well plates of Vero E6 cells overexpressing TMPRSS2 cells were inoculated at 37°C and gently rocked every 15 min for 1 h. After removing the inocula, plates were overlaid with 0.6% agarose containing 2% FBS. After 3 days, overlays were removed, plate were fixed with 20% paraformaldehyde (PFA) for 30 min and plaques were visualized by staining with 0.1% crystal violet. Viral titers were quantified as PFU per mL.

### Tissue processing

Routine mouse perfusion procedures were performed. Briefly, mice were anesthetized with ketamine/xylazine in accordance with IACUC guidelines. Mice were then transcardially perfused with phosphate-buffered saline (PBS) followed by freshly prepared 10% PFA in PBS. Both lungs and nasal cavity were post-fixed in 10% PFA in PBS overnight at 4°C. After 24 h, lungs were cryoprotected by immersion in 10% sucrose for 30 min, followed by 20% sucrose overnight. Lungs were subsequently infiltrated with 30% sucrose and kept at 4°C overnight. Subsequently, lungs were snap-frozen in tissue-freezing media. About 8–10 µM sections were obtained using an HM525 cryostat (Thermo Fisher Scientific) and stored at −80°C. The nasal cavity was fixed in zinc formalin, decalcified using EDTA, and embedded in Tissue-Tek O.C.T. Compound (SAKURA) before sectioning on a cryostat.

For lung histology, formaldehyde-fixed, paraffin-embedded tissue sections (4 µm each) were stained with H&E.

### Flow cytometric analysis and antibodies

After transcardial perfusion with PBS, lungs were removed and dissected into single lobes in a Petri dish containing PBS. All lobes were processed using a lung dissociation kit (Miltenyi Biotec, No. 130-095-927) to generate a single-cell suspension using an Octo Dissociator (gentleMACS, Miltenyi Biotech). Single cells were washed and blocked with 1 µg α-CD16/CD32 antibody for 15 min, then stained with the indicated antibodies on ice for 30 min. Cells were then fixed and permeabilized with Cytofix/Cytoperm Solution (BD Biosciences) for 24 h at 4°C. All flow cytometric data were acquired on an Aurora CS system (Cytek Biosciences).

The following antibodies were used for flow cytometry: CD45-eFluor605 (Invitrogen, 2251867), Live/Dead Blue (Invitrogen, 2674021), CD326 (Ep-CAM)-BV421 (Biolegend,118225), MHC II-BV510 (BioLegend, Clone-M5/114, 15.2), CD31-BV711 (BD, Clone-MEC 13.3), and Podoplanin (PDPN)-APC (Biolegend, Clone-8.1.1)

### Tissue staining and imaging

For antigen staining, frozen sections were warmed to room temperature for 10 min. Sections were placed in PBS for 10 min before a 10-min treatment with 0.1% Triton-X in PBS. After washing, sections were processed using a M.O.M mouse IgG blocking reagent (M.O.M immunodetection Kit, Vector Laboratories) to reduce nonspecific endogenous mouse IgG staining before primary antibody incubation. Sections were incubated for 4 h with primary antibody diluted in M.O.M diluent (M.O.M immunodetection Kit, Vector Laboratories). Sections were rinsed in PBS containing 0.1% Tween-20 and then incubated for 1 h at room temperature with fluorochrome-conjugated secondary antibodies (Thermo Fisher Scientific, 1:200). After incubation, sections were washed, sealed with Vectashield containing DAPI (Vector Laboratories). Images were obtained using a Zeiss LSM710 confocal microscope.

Primary antibodies reacting to KRT-8 (DSHB, TROMA-1, 1:1,000), Iba-1 (Wako, CAL0291, 1:200), cleaved-caspase 3 (R&D, MAB835, 1:200), PDGFrα (Santa Cruz, sc-398206, 1:100), proSP-C (Abcam, AB3786, 1:200), and RAGE (R&D System, MAB1179, 1:100), MHC II (BioLegend, Clone:M5/114.15.2, 1:200), SOX4 (Invitrogen, MA5-31424, 1:200), γH2AX (Novus Biologicals, NB100-74435, 1:200) , Cldn4 (Invitrogen, clone ZMD.306, 1:200), LGALS3 (Cedarlane, CL8942AP, 1:100), and CDKN1A (p21) (BD Biologicals, AB-396414, 1:200) were used.

### Quantification of cell skeleton

TdTomato^+^ cell morphology was quantified using a skeleton analysis method as previously described (17). Briefly, confocal images were acquired and tdTomato^+^ cells were enhanced to allow visualization of all tdTomato^+^ processes, followed by noise de-speckling to eliminate single-pixel background fluorescence (ImageJ/FIJI, NIH). The processed image was converted to a binary (black and white) format (Fig. S4a, middle panel), with cytoskeletal structures represented as white lines on a black background (Fig. S4a, right panel). The “Analyzeskeleton” function on ImageJ or Fiji was then applied to all skeletonized images to collect data on the number of endpoints per frame and process length. These data were used to assess the branch morphology of tdTomato^+^ cells.

### Quantification of protein expression (ImageJ/Fiji, NIH)

Mean fluorescence intensity (MFI) of Iba-1 expression was quantified using ImageJ or Fiji software (NIH, Bethesda, MD, USA). Briefly, images were opened in ImageJ. Regions of interest (ROIs) were defined and selected. The amount of Iba-1 staining in ROIs was calculated using Fiji. The Iba-1 MFI from each image was averaged for each animal and compared between experimental groups each of which contained three animals. Three or more images per section and >3 sections per animal were analyzed. For quantification of Iba-1/Venus ratio, MFI value of Iba-1 was divided by the MFI value of Venus in the same ROI.

### Image acquisition, processing, and quantification

Images were captured using a Zeiss LSM 710 confocal microscope system with 20×, 40×, or 60× objectives. Cells were manually counted based on immunohistochemical markers and DAPI. Images were processed using ImageJ or Zen software. In all experiments of image quantification, each experimental group had at least five mice and we analyzed 10–20 sections per mouse.

### Statistics

Data were analyzed using two-tailed Student’s *t* tests. A *P* value of <0.05 was considered significant. Mann-Whitney *U* tests were used to analyze differences in means when samples were nonparametric. Results are represented as means ± SEM. *P ≤ 0.05, **P ≤ 0.01, and ***P ≤ 0.001.

## SUPPLEMENTAL MATERIAL

Supplemental Figures mbio.03693-24-s0001.pdf Figures S1 to S5.

ASM does not own the copyrights to Supplemental Material that may be linked to, or accessed through, an article. The authors have granted ASM a non-exclusive, world-wide license to publish the Supplemental Material files. Please contact the corresponding author directly for reuse.
