# Supplementary material for: Cells that survive acute SARS-CoV-2 infection contribute to inflammation and lung regeneration in mice
Source: mBio. 2025 Jan 29;16(3):e03693-24. doi: 10.1128/mbio.03693-24 (PMC11898547; doi:10.1128/mbio.03693-24)
Supplement: Supplemental Figures — Figures S1 to S5. [file mbio.03693-24-s0001.pdf]

Figure S1

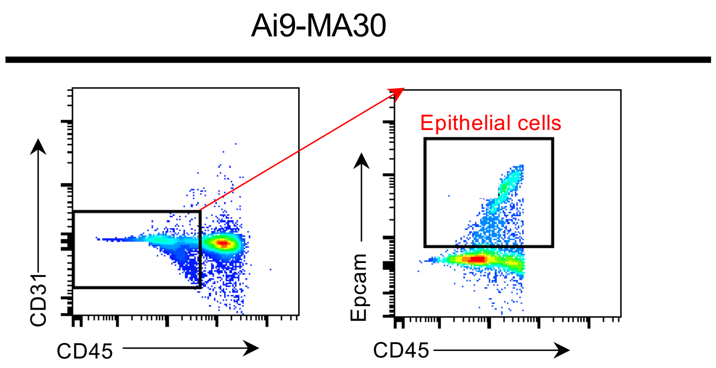

Figure S2

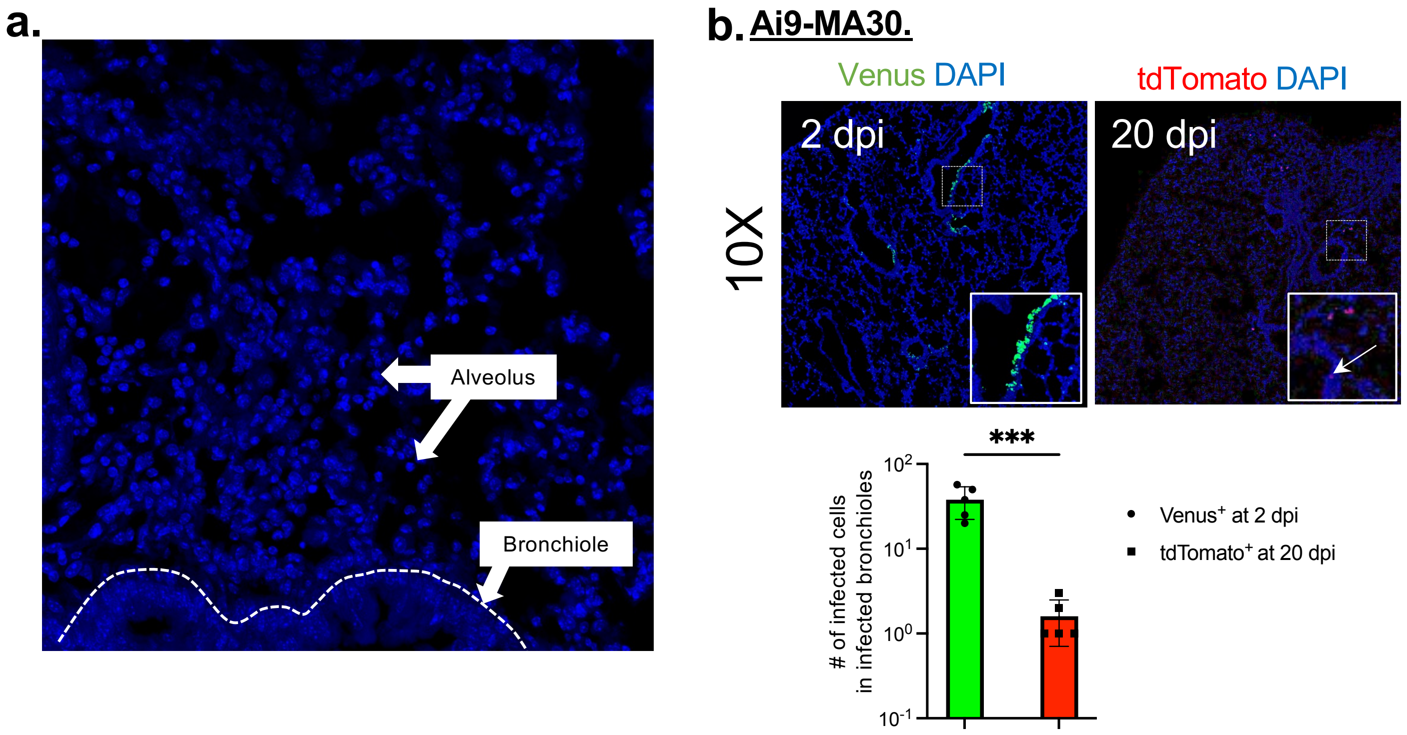

Figure S3

a.

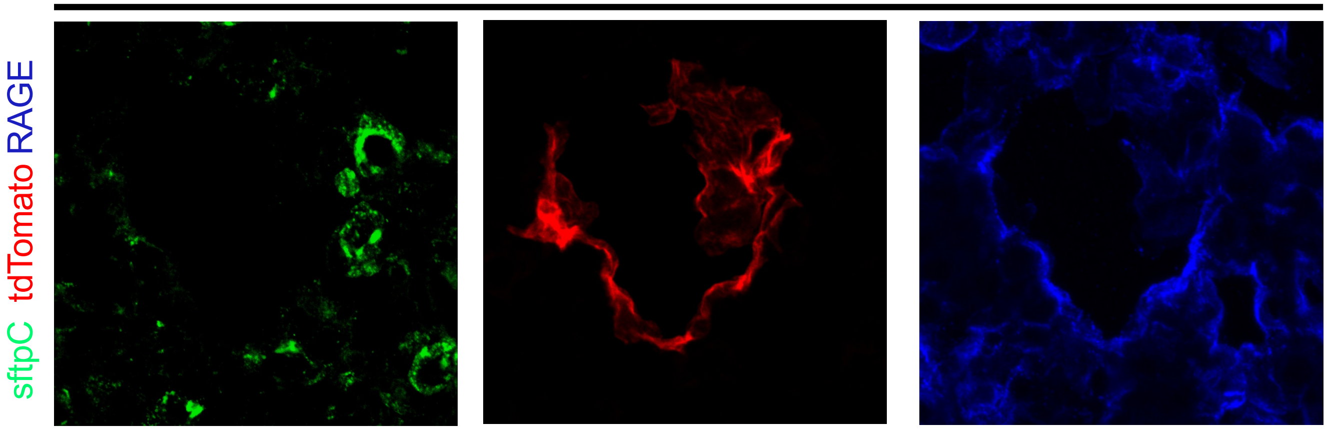

b.

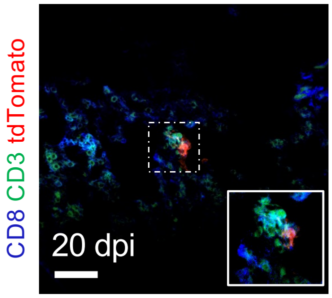

Figure S4

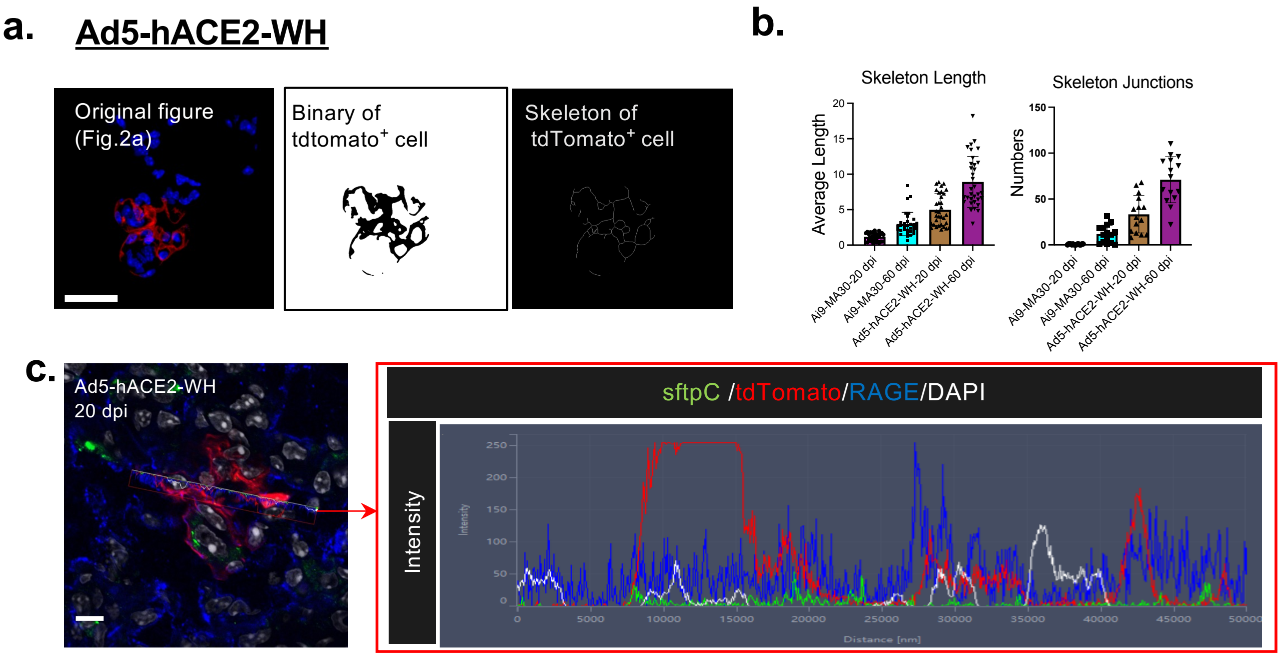

Figure S5

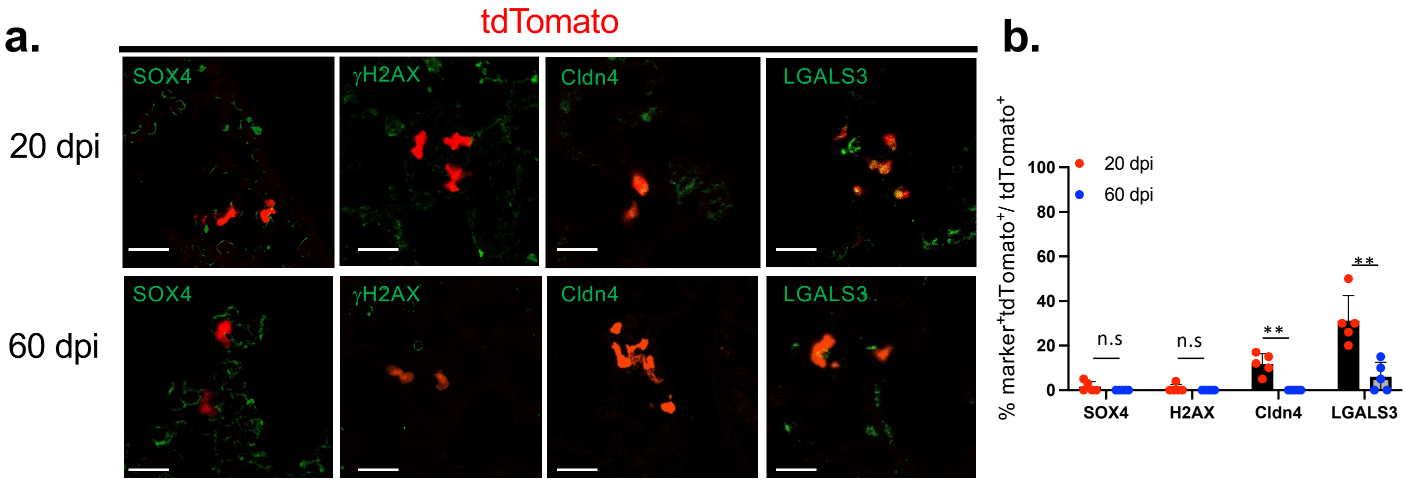

Supplementary Figures

Figure S1. Epithelial cell analysis at 2 days post-infection

The gating strategy for epithelial cell analysis, as referenced in Figure 3a, is shown.

Figure S2. Comparison of lung sections at 2 dpi versus 20 dpi in rSARS2-MA30-V2C-infected Ai9 mice

a. Identification of bronchioles and alveoli in panel shown in Figure 3d (blue, DAPI). Scale bar, 50 μm.
b. Numbers of infected cells in the bronchioles at 2 dpi (left, Venus+) and at 20 dpi (right, tdTomato+). Only bronchioles with infected cells were chosen for further analysis. Average number of infected bronchiolar cells and surviving bronchiolar cells are summarized. Only a few infected cells in the bronchioles survived the acute infection. P=0.009 (two-tailed, unpaired t test). n=5. Nuclei (blue, DAPI). Scale bar, 100 μm.
   Statistical analysis was performed using two-tailed unpaired, non-parametric t-test (Mann-Whitney test); ***P<0.001.

Figure S3. AT1 cells in rSARS2-MA30-V2C-infected Ai9 mice are immunoresponsive and differention.

a. Single channels for tdTomato (red), sftpC (Green) and RAGE (blue) corresponding to the merged image shown in the right panel of Figure 3b.
b. Sections from lungs of rSARS2-MA30-V2C-infected Ai9 mice at 20 dpi were stained for CD3 (green) and CD8 (blue). Higher magnification images of selected areas are shown. Scale bar, 20 μm.

Figure S4. Previously infected AT1 cells in Ad5-hACE2/Ai9 mice exhibit elongated processes.

a. tdTomato expression of cell shown in Fig. 2a (left panel, boxed) was converted to a binary image and then skeletonized. Skeleton length and junctions were analyzed for statistical significance using ImageJ. This method was used to quantify the branches of tdTomato+ cells in Ad5-hACE2/Ai9-transduced mice infected with rSARS2-WH-V2C and rSARS2-MA30-V2C-infected Ai9 mice at 20 dpi and 60 dpi. Data are summarized in Figure 5c and S2b. Scale bar, 50μm.

b. Comparison of skeleton length and junctions of tdTomato+ cells at 20 dpi and 60 dpi in rSARS2-WH-V2C-infected Ad5-hACE2/Ai9 mice and rSARS2-MA30-V2C-infected Ai9 mice (from Figure 5c).

c. Lung sections from infected Ad5-hACE2/Ai9 mice were analyzed for expression of AT1 and AT2 markers and tdTomato. tdTomato+ cells expressed only AT1 markers (RAGE, blue). (Right) Cells along the indicated line were analyzed for marker expression using ZEN Software (Zeiss) as described in the Methods section. Scale bar, 10 μm.

Figure S5. Expression of PATS markers in previously infected cells at 20 dpi and 60 dpi.

a) Sections from lungs of rSARS2-MA30-V2C-infected Ai9 mice were analyzed at 20 dpi and 60 dpi for SOX4, γH2AX, Cldn4 and LGALS3 expression. Scale bar, 20 μm.

b) Portion of indicated targets positive cells in all tdTomato+ cells. n=5 mice.
